# Physiological Role of Orexin/Hypocretin in the Human Body in Motivated Behavior: A Comprehensive Review

**DOI:** 10.7759/cureus.34009

**Published:** 2023-01-20

**Authors:** Rahul Singh, Dalia A Biswas

**Affiliations:** 1 Department of Neurophysiology, Jawaharlal Nehru Medical College, Datta Meghe Institute of Medical Sciences, Wardha, IND; 2 Department of Physiology, Jawaharlal Nehru Medical College, Datta Meghe Institute of Medical Sciences, Wardha, IND

**Keywords:** thermogenesis, dora, obesity, brown adipose tissue, drug abuse, homeostasis, narcolepsy, hypothalamus, hypocretins, orexin

## Abstract

Neurohormones are neurosecretory materials released by neurosecretory cells that serve both as neuromodulators in the brain and spinal cord and as circulating regulatory hormones. They serve a wide range of functions, including homeostasis, development, and modulation of neuronal and muscle activity. In the hypothalamus, neurohormones called hypocretins are created that were discovered in the late nineties. Orexin receptors (OXRs) have been shown to enhance synaptic signaling in the central nervous system at the cellular level. The orexins improve stimulated neural activity in the hippocampus, which, in turn, aids with spatial memory, learning, and mood. They present themselves as mediators for the hypothalamic functions. They have been shown to regulate sleep-wake cycles, arousal mechanisms, addiction, sympathetic nerve activity (SNA), blood pressure, and thermogenesis. Its role in storing brown adipose tissue has implications for thermal homeostasis. The significant role of orexins is seen in tumorigenesis when orexin A (OrxA) and orexin B (OrxB) induce apoptosis in fast-growing tumor cells. Orexin-null subjects show clinical narcolepsy, indicating that orexins were responsible for keeping them awake. Orexin microinjections in mice brains stimulated increased physical activity, thus possibly countering diet-induced obesity. Physical activity significantly increased plasma orexin-A levels, which facilitated the process of energy homeostasis. The amount of adrenocorticotropic hormone (ACTH) increases in stress conditions, which further facilitates the release of the stress hormone cortisol. No increase in the ACTH hormone is seen in stressed mice administered with orexin receptor 2 (OX2R) antagonists thus showing orexin's role in stress reaction. As a result of linking hypocretin/orexin to various physiological procedures, increased research into the medicinal potential of drugs targeting these receptors is emerging. We summed up in this review the recent advances in our understanding of how orexin and its receptor system play an essential role in clinical and pathological functions. This research summarizes a new area for research in human medicine, providing the possibility of controlling a vast array of physiological functions through intra-cerebroventricular injections of a single neuropeptide.

## Introduction and background

The hypothalamus is a part of the brain weighing around 4 grams but serving essential functions. Yet, this region has highly preserved neuronal circuitry that regulates whole life processes. The hypothalamus has many functions, among which controlling food intake and energy balance are the hypothalamus' principal functions. All of its major functions are summarized in Table 1. 

**Table 1:** The major hypothalamic roles and functions [1]

S. No.	Roles	Major Functions
1.	Energy	Thermoregulation, fever symptoms
2.	Metabolic process	Metabolic regulation, food intake, digestion, energy expenditure, homeostasis, excretion
3.	Fluid Balance	Balance of electrolytes, fluid utilization, fluid absorption
4.	Wake-Sleep cycle	Diurnal variations, emergency reactions, and cycles to environmental stresses
5.	Reproduction	Replication via mating, hormone regulation, pregnancy, parturition, and lactation

Orexins, also called hypocretins, are excitatory neurohormones identified at the end nineties by two separate investigatory teams. Due to their capacity to promote food intake and regulate metabolism, Sakurai et al. (1998) used chromatography to identify these new neuropeptides and gave them the names "orexins" (Orx-A and Orx-B), which come from "orexis" in Greek, which means appetite. Proteolytic processing of a common precursor led to the discovery of two novel neuropeptides, orexin-A and orexin-B, that combine and stimulate two nearly associated orphan G protein-coupled receptors (GPCR), the orexin receptors 1 and 2. Molecular biological techniques were used to identify these new neuropeptides. The names hypocretins (Hcrt1 and Hcrt2) were given to them because they are produced by the hypothalamus and because they share a significant amount of amino acids with secretin [2].

Orexin-A and Orexin-B are two related peptides that share no structural features with any recognized kinds of regulating peptides. Nerves that express prepro-orexin messenger RNA and immunoreactive orexin-A are located in the lateral and posterior hypothalamus of the adult rat brain. These peptides lead to an increase in food intake when given centrally to rats. Fasting increases prepro-orexin mRNA levels, pointing to a possible physiological role for the peptides as mediators in the central feedback loop that controls feeding behavior [3]. As a result of its interactions with receptor 1 (OX1R) and receptor 2 (OX2R), orexin is involved in the control of eating behavior by activation of the downstream signaling pathway [4].

**Figure 1:**
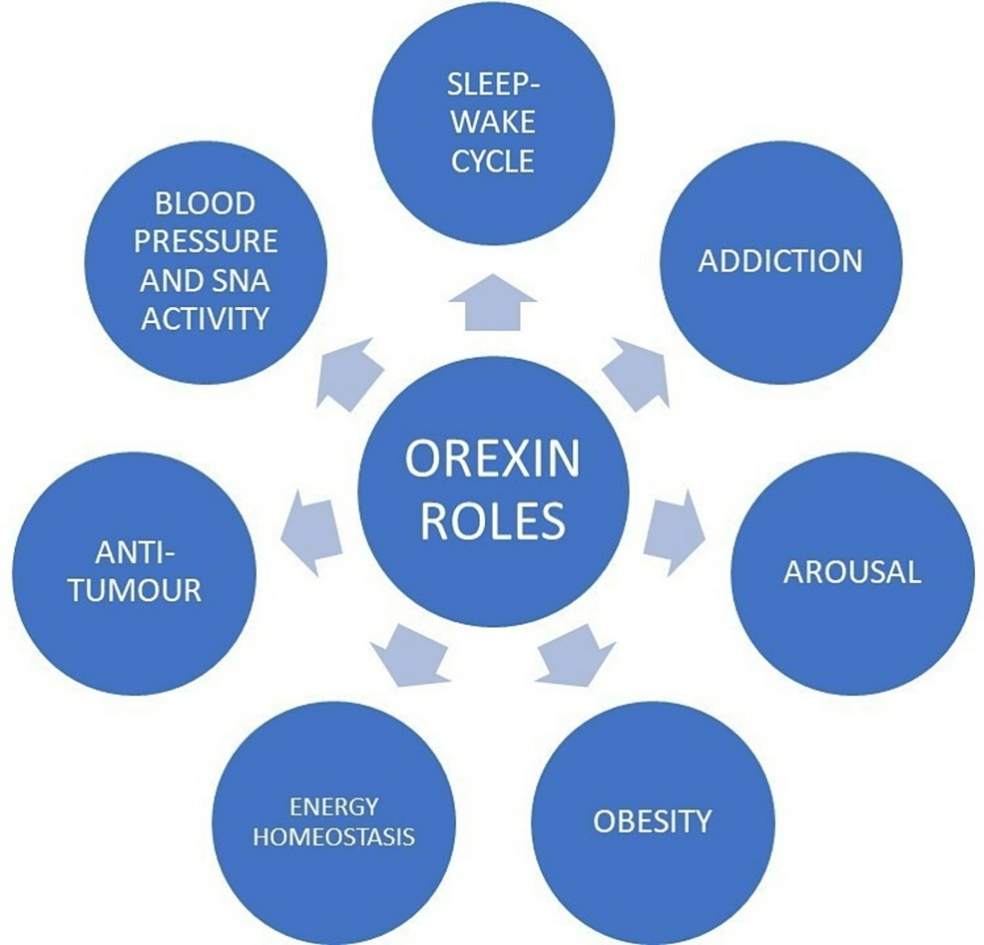
Roles of Orexin as seen through the flowchart [4]

A sleep disease, Narcolepsy type - 1 is caused by the hypocretin signal being compromised, either by gene deletion of the prepro-orexin or orexin 2 receptor or through the excision of hypocretin nerve cells [5]. The main function of orexin in the central nervous system (CNS) was to keep people awake [6]. One of the hypothalamus neuropeptide families with the broadest range of effects, orexins have now been understood to play a pivotal role in commanding a comprehensive range of physiological functions, including rest and recovery, energy consumption, pain, cardiovascular health, and neuroendocrine function [7]. “Hypocretin” denotes the gene part, and "orexins" for the protein part [8]. In the human central nervous system, the loss of orexin neurons and the lack of orexin production result in cataplexy and narcolepsy (Narcolepsy type I). Orexin receptor-targeting compounds, mostly antagonists, with the ability to control the wake-sleep cycle, have been developed by the pharmaceutical industry and academic labs as a result of the effect of orexin on sleep regulation [9]. Research has also shown that g-protein-coupled receptors (GPCRs) ORX1 and ORX2 receptors play a significant part in tumor formation, establishing their participation in cancer onset, progression, and metastasis [10]. The central regulation of sympathetic nerve activity (SNA) and blood pressure is one of the many processes the orexins manage. Recent research has studied the architecture of orexin-producing neurons in connection to brain areas that control blood pressure and SNA. Figure 1 shows the various roles of orexin, which could have further implications in pharmacological aspects [4]. 

Moreover, there have been several in vivo functional studies demonstrating the orexin system's role in regulating both blood pressure and SNA [11]. When experimental rats had a history of cocaine and alcohol, a selective orexin-1 receptor antagonist reduced cocaine intake and motivation, but not when rats had a restricted history of cocaine alone [12]. This hints toward a possibility of orexins being used to counter drug abuse. Thus, through this review, we seek an impetus for future explorations in the orexin system in the neuropharmacology field through novel disease diagnosis and treatment methods.

## Review

Methodology

We undertook a systematic search through PubMed, CENTRAL, Medline, Google Scholar, and the Web of Science to look for relevant studies in January 2022. We employed such terms to probe or look for: orexin, hypothalamus, and homeostasis ((("orexin*" [Title/Abstract]) OR ("Orx*" [Title/Abstract]) OR ("orexin*" [MeSH Terms])) AND (("hypothalamus" [Title/Abstract]) OR ("hypothalamus" [MeSH Terms])) AND (("homeostasis" [Title/Abstract]) OR ("homeostasis*" [MeSH Terms])). Key references were searched from the bibliographies of relevant studies, which were conducted by two reviewers over seven months from January 2022 to July 2022. All literature was vetted by title to ensure they were suitable. The search was updated in October 2022.

**Figure 2:**
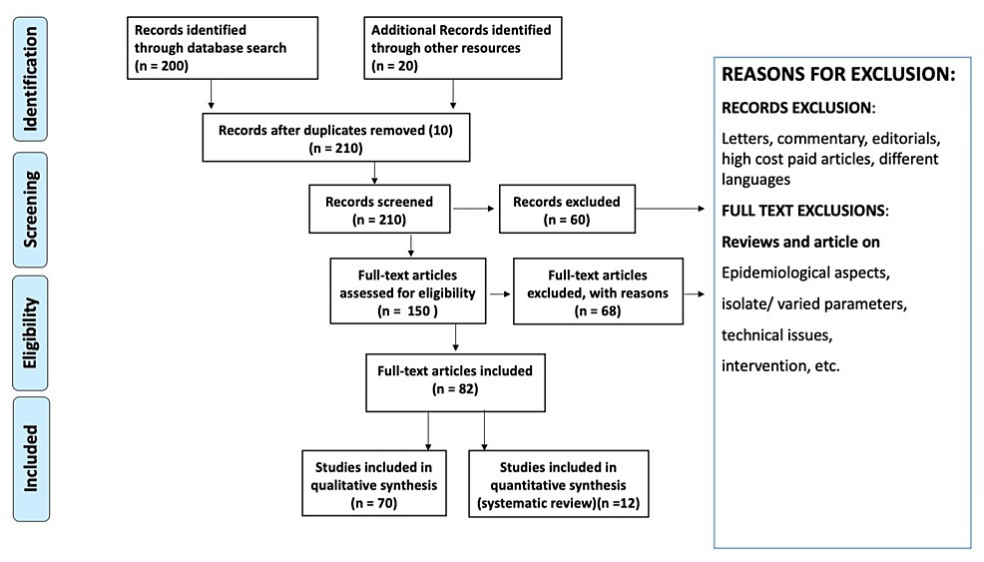
Diagram showing the research methodology by PRISMA method PRISMA: Preferred Reporting Items for Systematic Reviews and Meta-Analyses

One reviewer independently monitored the retrieved studies against the inclusion criteria, in the beginning, based on title and abstract and then on full texts. Another reviewer also reviewed approximately 20% of these studies to validate the inclusion of these studies. We excluded incomplete, non-English and duplicate articles. The identified studies were initially screened using titles and abstracts. Records that passed the full-text review were subjected to the second round of selection. The procedure of selecting studies is depicted in Figure 2. To compare studies, we compiled the following information for each one: first author's name, year of publication, research population, age, sex, orexin applications, the outcome of utilizing orexin antagonists, main results, and conclusion.

Discussion

The hypothalamus is situated near the bottom of the brain, flanked in both directions by the optic tract and the crus cerebri and bounded anteriorly by the posterior perforated substance. In the sagittal section of the brain, it is surrounded by the lamina terminalis in the front, the floor of the third ventricle from the optic chiasma to the posterior perforated substance in the lower half, and the hypothalamic sulcus in the upper half. The hypothalamus may be roughly divided into the periventricular, intermediate, and lateral zones. It is subdivided into four regions, namely the preoptic, the supraoptic, the tuberal, and the mammillary regions. The entire hypothalamus sees several aggregations of neurons scattered around called nuclei. They are preoptic, supraoptic, paraventricular, suprachiasmatic, anterior, arcuate, dorsomedial, ventromedial, posterior, mammillary, and lateral nuclei. Several functions of the body show a cyclic variation over the day. The most recognizable is the sleep-wake cycle. The suprachiasmatic nucleus is believed to play an important part here. Lesions of the hypothalamus disturb the sleep-waking cycle.

Neurons in the spinal cord that can detect heat or cold send their signals to separate regions of the pons called the lateral parabrachial nucleus, which is sensitive to heat and cold, respectively. The parabrachial nucleus acts as a relay station for the transmission of a variety of visceral sensory transmission of information from the brainstem and spinal cord to the hypothalamus, as well as for the retransmission of hypothalamic feedback to the groups of cells responsible for autonomic reflexes and motor control. There are substantial connections between the medial preoptic nucleus and the ventromedial nucleus, and these connections are assumed to regulate sexual behavior's motor and autonomic rhythms. Neurons in the ventromedial nucleus of male mice have been demonstrated to activate cFos in two distinct but overlapping populations: during hostile confrontations (by a male invader) and sexual interaction (with female mice) [1].

Role of the Hypothalamus in the Orexinergic System

Orexins are neuropeptides that are yielded from a collection of neurons in the lateral hypothalamic region (LHA) of mammals. These neurons are located in the perifornical, posterior, lateral, and dorsomedial nuclei [4]. Pre-pro-orexin is a 131-residue peptide whose mRNA has a gene responsible for it on chromosome 17 in humans [13]. The C-terminal region of Orx-B is identical to that of Orx-A, but the N-terminal section displays considerable diversity. The highly preserved structures of these hormones might be connected to the relevance of their activities [14].

Typically, neuronal depolarization via blockage of K+ channels is the result of orexin receptor activation, and Na+ increased Na+/Ca2+ influx owing to activated Na+/Ca2+ non-selective cation exchangers and ion channels whose signaling cascades are yet not fully understood [4,15,16]. Many lines of evidence lead to Gq activation playing a crucial part in OXR signaling; however, most of this is extrapolated, and further work is needed to confirm this in native cells. OXRs have been shown to enhance synaptic signaling at the cellular level in the central nervous system (CNS) [16]. In the CNS, OXRs are widely expressed, albeit not everywhere. Their expression is particularly strong on monoaminergic and cholinergic neurons in the brain stem and brain regions associated with arousal, cognition, stress, and reward, demonstrating distinct patterns of distribution that are consistent with the specific physiological functions of OX1R and OX2R [4,6,15-18]. Orexin neurons in humans are believed to number between 50,000 and 80,000 neurons, all of which are located in the hypothalamus and provide signals to several areas of the brain and the whole spinal cord. The brain stem's monoaminergic and cholinergic nuclei receive the bulk of the nervous system's projections, as well as the basal forebrain, locus coeruleus, dorsal raphe nucleus (DRN), tuberomammillary nucleus, and ventral tegmental area [4,19]. It has been established that interactions between orexin and the paraventricular thalamus have a role in regulating sleep, reward addiction, cognition, and stress. Addiction and sympathetic function are influenced by the Orx-PVN pathway, which is activated by a high concentration of orexin neuron projections that reach the paraventricular nucleus (PVN). Additionally, orexin projections to the ventral pallidum (VP) are associated with hedonic valence processing, and those to the insular cortex may play a role in amplifying hypocretin signals in the course of this process [6].

Role of Orexin in Sleep Regulation

Sleep disruption and short sleep duration were related to increased risk for depression and recognizable medical mistakes in a study of first-year medical residents [20]. Wakefulness is facilitated by the discharge of glutamate and other excitatory peptides from synapses in various brain regions, which are engaged in modulating levels of arousal from hypothalamic orexin-producing neurons. In contrast, hypocretin/orexin antagonists are used to treat insomnia and other sleep disorders by facilitating sleep by blocking the awake urge mediated by the hypocretin/orexin system [21]. First identified as sleep-wakefulness regulators due to the OX2R mutation seen in narcoleptic dogs, orexins have since undergone extensive study [22].

According to the "flip-flop model" of sleep- and wake-promoting brain areas, aminergic regions stimulate the cortex directly and suppress the activity of sleep-inducing neurons in the ventrolateral preoptic nucleus to keep you awake [23]. Despite being the first dual orexin receptor antagonist (DORA) to be demonstrated to shorten sleep latency across animals and to reduce the waking time after sleep onset (WASO) in people, almorexant's clinical development was halted due to its hepatoxicity [24,25]. Suvorexant binds to hypocretin 2 (HCRTR2)/OX2R, where it stabilizes an extracellular salt bridge network and prevents transmembrane helix movements essential for receptor activation [26]. Polyphasic sleep is common in laboratory mice, and this suppression of orexin receptor signaling aids napping by reducing the awake-stimulating action of the orexin system. This results in more frequent transitions to non-rapid eye movement (NREM) and rapid eye movement (REM) sleep [27]. Several behaviors in hypocretin 1 (HCRTR1)/OX1R knockout (KO) mice are altered, including minor sleep disturbance, nervousness, depression-like behavior, and a hyperactive alarmed response, as well as reduced locomotor activity, prepulse inhibition, and societal contact [28]. Sleep-wake state disruption and cataplexy-like occurrences define the narcoleptic phenotype seen in HCRTR2/OX2R lacking mice [29]. The intranasal delivery of orexin-A has been shown to increase alertness, mitigate the adverse effects of sleep deprivation on learning and memory, and help narcoleptic patients. These effects have been observed in rhesus macaque monkeys and human subjects [30,31].

Role of Orexin in Digestive Carcinomas

According to GLOBOCAN, cancer, despite ongoing improvements in the treatment arsenal, is the world's second largest killer globally [32]. Cancer of the digestive tract, which includes the colon, pancreas, liver, stomach, and esophagus, is the second most common kind of cancer after lung cancer [33]. In 2004, neuropeptides, hormones, and orexins were among the 26 peptides whose effects were examined on the expansion of colorectal cancer (CRC) cell line HT-29. OrxA and OrxB were the only peptides that significantly slowed down the expansion of tumor cells, whereas the great majority had no impact. The development of HT29-D4 human colon cancer cells is stifled by orexins because they cause apoptosis by releasing cytochrome c from mitochondria and activating caspases. [34]. CRC patients were given 5-fluorouracil for therapy (5-FU). As the HT-29 colon cancer cell line became resistant to 5-FU, it was discovered that OX1R was consistently expressed and that hypocretins caused a pro-apoptotic impact in these cells. Drug-resistant cancer cells maintained their sensitivity to the reaction. In mice with pre-existing xenografted tumors (tumor volume of around 150-200 mm^3^), therapy with OrxA resulted in a quick and robust regression of the tumors; reverse the growth of tumors was seen, proving that OrxA was successful in diminishing confirmed malignancies [35]. Thus, the role of orexin in cancers calls for further research in the field, as it might prove to be a probable cure.

Role of Orexin in Obesity

In addition to warding off obesity and the mental fogginess that comes with aging, regular exercise may boost overall health. Many people either don't want to or refuse to carry it out and are looking for ways around it. It is important to go down to a healthy weight and stay there [36]. Independent of both physical activity and sedentary time, elevated orexin-A levels were related to a greater risk of becoming overweight or obese [37]. Studies have found that levels of orexin-A control the amount of glucose in the blood and CSF [38]. Sprague-Dawley rats had orexin-A injected into three brain projection locations, leading to increased activity and decreased sluggish behavior [39]. Diet-induced obesity is not as common in mice when the orexin gene has been highly expressed. Because of this, orexin signaling may favorably control food intake and wakefulness, but additionally, physical exertion and energy use during rest lead to weight gain resistance [40]. Reduced sensitivity of its receptors in adipose tissue is linked to low levels of circulating OrxA in obese males, as has been widely characterized [41]. Orexin signaling is downregulated in an obese person [42]. Food deprivation, whether in animals or people, causes an increase in the production of the hormone orexin as well as its receptor mRNA and peptide [36,41]. When glucose levels are too high, orexin neurons are immediately shut off and serve as instruments for adaptive glucose sensing. This suggests that hyperglycemia makes orexin signaling weaker [43]. When injected into the brains of rats, orexin-A, specifically the rostral lateral hypothalamus, the mice lost weight, became more active on their own accord, burned more calories, and ate less [44]. Thus, knowledge of orexin concentrations might be used as a novel treatment approach to managing weight by also changing levels of physical activity and energy consumption.

Role of Orexin in Thermal Homeostasis

It has been shown that the lateral hypothalamus plays a crucial part in controlling alertness, eating habits, prompted actions, and thermogenesis [45]. Orexin neurons are involved in eating, sleep-wake cycles, spontaneous physical activity (SPA), muscle tone, motor activity, energy expenditure, and information of other physiological processes all converge here [46]. In humans, the manifestations of spontaneous physical activity include fidgeting, standing, and walking [47]. Certain persons do, while others do not enhance their SPA and NEAT (non-exercise induced thermogenesis) to resist obesity when overfed, demonstrating that SPA and NEAT variations in different individuals play an important role in energy balance [48]. Physical activity raises plasma orexin-A levels, which stimulates the SNA and the process of energy homeostasis [36,49,50]. Hyperthermia was elicited in fasted mice after central administration of Orx-A [51]. The hyperthermic effects of Orx-B have been documented in another investigation [52]. The absence of orexin disrupts energy homeostasis, as seen in orx-null mice [7]. An oral dose of DORA reduces SPA for eight hours and core body temperature for four hours [53]. The treadmill-running exercise-induced increase of core body temperature (CBT) was attenuated after DORA administration, indicating further a function for orexin in thermoregulation during exercise [54]. Humans are mammals, and most mammals engage in non-shivering facultative thermogenesis that occurs in the brown adipose tissue (BAT) [55]. Efforts aimed at bolstering the function of the orexin-serotonin axis in thermogenesis the orexin-serotonin axis is responsible for promoting sympathetic outflow and directing metabolic processes; increasing sympathetic activation of BAT is another effect of orexin injection into the DRN neurons [56,57]. These findings indicate that orexin can boost thermogenesis via interacting with DRN serotonin, which increases sympathetic outflow and decreases parasympathetic output [58,59]. More profound research is thus needed to determine if the effects of orexins on BAT are unique to mice or else a potential gap in the pathway of human therapeutic intervention.

Role of Orexin on Cardiovascular System and Blood Pressure

A recent study has shown that the dorsal motor nucleus of the vagus nerve is linked to orexin neurons via direct neural pathways [60]. The rostral ventrolateral medulla is another target of orexin neurons (RVLM). This link triggers adrenaline production in response to hypoglycemia and other stressors [61]. In Sprague Dawley (SD) rats, 14 days of foot shocks and loud sounds create a stress-induced hypertension rat (SIHR) model, altering NOS (nitric oxide synthase) isoform expression in the RVLM, a crucial area mediating the cardiovascular effects of orexin A [62]. Both hypertensive and normal rats showed a considerable rise in blood pressure and heart rate after receiving microinjections of orexin-A into the RVLM [63]. Arterial pressure was lowered in both hypertensive and control rats when given high doses of almorexant, and the drug also dampened the animals' pressor and tachycardic reaction to stress. The pressor response to stress was unaffected by the blocking of OX1R, although the blood pressure and heart rate following stress exposure were reduced [64]. The significance of orexin in stress reaction is even more elaborate, as seen in the study when in stressed mice administered with OX2R antagonists, no increase in the ACTH hormone was seen [65]. The researcher Huang demonstrated that an OX2R antagonist mitigated the elevation in blood pressure and heart rate induced by phenotypically localized orexin-A injections into the RVLM, but an OX1R antagonist merely mitigated the elevation in blood pressure [66]. Thus, OX1R is the one that has a major role in regulating sympathetic nerve activity (SNA) and, subsequently, blood pressure. Further research on the topic would be beneficial in additional human physiological applications and preventive cardiovascular medicine.

Role of Orexin in Addiction and its possible use in countering Drug Abuse

Opioids were shown to inhibit hypocretin/orexin neuron activity, whereas blocking μ ­-opioid receptors increased hypocretin (Hcrt) neuron activity in research. When morphine is applied to Hcrt neurons, the neurons' future excitatory responses to Hcrt are dampened [67]. However, recent studies demonstrate that Hcrt unit activity is significantly increased in intact rats when morphine is administered systemically [68]. These Hcrt cells release glutamate [69] and corelease dynorphin [70]. The two neuropeptides perform polar opposite roles when it comes to the reward circuit, which may be seen in things like drug seeking, impulsivity, and self-administration of narcotics and alcohol [71,72]. The brains of non-addict individuals only release Hcrt when they are doing something pleasurable, unlike when they are startled by pain or upset [73]. From a clinical point of view, the difficulty of many opiate addicts to safely quit opioid usage is the most crucial issue among opiate addicts [67]. In both human heroin users and mice given morphine continuously, researchers observed a dramatic multiplication in the number of Hcrt-generating neurons [68]. Further, in studies using alcohol, cocaine, opioids, and nicotine, OX1R antagonists reduced addictive tendencies. Additionally, orexins' role in regulating drug withdrawal behaviors has been shown, notably in the context of opioid withdrawal [74]. Morphine removal boosted orexin gene transcription and neuronal activity in mice [75]. Moreover, it has been demonstrated that blocking OX1R (OX1R antagonism) decreases the increases in Fos expression that result from withdrawal in several brain regions [76,77]. Pre-treatment of high alcohol-preferring rats with the OX1R antagonist decreased their alcohol consumption, self-administration, and reinstatement in response to cues [78]. The orexin system's involvement in both reward and stress/arousal is consistent with this view. Stress-induced cravings for alcohol and cocaine can be effectively suppressed by administering an OX1R antagonist [79-81]. Additional preclinical research is therefore needed to clarify how these drugs could be useful in the clinical setting of prescription opioid use disorder (OUD) [82]. Prescription opioid use disorder is a complex disease, and one novel method might be to block the signaling of the Orx molecule, as is suggested by the current literature. 

Limitations

Due to the nature of the research questions and the limited usage of Cureus, PubMed, Scopus, and Web of Science database search results, this study relied mostly on qualitative research methods. Some quality content was; however, not accessible due to being high-paid articles, while some were in different languages. This research's review did not, for the most part, provide statistically meaningful results. Rather, the analysis led the team to an assortment of varied case studies from which to conclude. While the case studies were extensively studied and confirmed, the sample size of the case studies limits the validity of the conclusions. It is likely that in the future, other functions not uncovered in this study will emerge.

## Conclusions

New orexin research, data, and information have emerged in recent years. Our understanding of this system has been revised, increased, and clarified. Considering the extensive network nature of this system, potential future investigation and unique data may reveal both the direct and indirect actions of how the orexin system controls cognition, energy levels, reward attitude, mood, and appetite. The significance of their tangential impacts uncovered the fact that the orexin system has great potential as the therapeutic agent for a broad array of human diseases across the spectrum, from narcolepsy to digestive cancers to obesity. Learning more about how the different parts of the brain connect anatomically and functionally and how neural calculations are performed will not only help us get a better grasp of how the orexin system works but also contribute to the discovery of novel therapies for conditions as diverse as metabolic, locomotory and neurological disorders. Further research on human subjects to confirm if they are affected in the same way by orexin microinjections and orexin null species as rodents need to be done so as to clear the research gap in therapeutic medicine. We have also argued that DORAs reduce drug desire and enhance sleep outcomes, thus could be beneficial in the treatment of substance use disorders (SUDs).
